# Co-Expansion of Cytokine-Induced Killer Cells and Vγ9Vδ2 T Cells for CAR T-Cell Therapy

**DOI:** 10.1371/journal.pone.0161820

**Published:** 2016-09-06

**Authors:** Shou-Hui Du, Zhendong Li, Can Chen, Wee-Kiat Tan, Zhixia Chi, Timothy Weixin Kwang, Xue-Hu Xu, Shu Wang

**Affiliations:** 1 Department of Biological Sciences, National University of Singapore, Singapore, 117543, Singapore; 2 Institute of Bioengineering and Nanotechnology, Singapore, 138669, Singapore; 3 Tessa Therapeutics, Pte Ltd., Singapore, 239351, Singapore; 4 Department of Gastrointestinal Surgery, the Third Affiliated Hospital of Guangzhou Medical University, Guangzhou, 510150, China; Universita degli Studi di Palermo, ITALY

## Abstract

Gamma delta (γδ) T cells and cytokine-induced killer (CIK) cells, which are a heterogeneous population of T lymphocytes and natural killer T (NKT) cells, have been separately expanded *ex vivo* and shown to be capable of targeting and mediating cytotoxicity against various tumor cells in a major histocompatibility complex-unrestricted manner. However, the co-expansion and co-administration of these immune cells have not been explored. In this study we describe an efficient method to expand simultaneously both CIK and Vγ9Vδ2 T cells, termed as CIKZ cells, from human peripheral blood mononuclear cells (PBMCs) using Zometa, interferon-gamma (IFN-γ), interleukin 2 (IL-2), anti-CD3 antibody and engineered K562 feeder cells expressing CD64, CD137L and CD86. A 21-day culture of PBMCs with this method yielded nearly 20,000-fold expansion of CIKZ cells with γδ T cells making up over 20% of the expanded population. The expanded CIKZ cells exhibited antitumor cytotoxicity and could be modified to express anti-CD19 chimeric antigen receptor (CAR), anti-CEA CAR, and anti-HER2 CAR to enhance their specificity and cytotoxicity against CD19-, CEA-, or HER2-positive tumor cells. The tumor inhibitory activity of anti-CD19 CAR-modified CIKZ cells was further demonstrated *in vivo* in a Raji tumor mouse model. The findings herein substantiate the feasibility of co-expanding CIK and γδ cells for adoptive cellular immunotherapy applications such as CAR T-cell therapy against cancer.

## Introduction

Adoptive immunotherapy for cancer has emerged as a fast developing field that shows great promise in recent clinical trials. This therapy approach involves the isolation of immune cells, *ex vivo* cell expansion and reinfusion of the expanded lymphocytes into patients to treat cancer. Successful examples of adoptive immunotherapy to eradicate tumor cells in patients with malignancies include expansion and transfusion of autologous tumor-infiltrating lymphocytes (TIL), T cell receptor (TCR)-modified T cells, and chimeric antigen receptor (CAR)-bearing T cells.[1] Besides conventional T cell subsets, many other types of immune cells, for example cytokine-induced killer (CIK) cells and gamma delta (γδ) T lymphocytes, have also been exploited for adoptive immunotherapy of cancer.[2–4]

CIK cells are lymphocytes *ex vivo*-generated with an initial priming with IFN-γ and anti-CD3 antibody (clone OKT3) followed by repeated stimulation with IL-2.[4,5] CIK cells can be quickly expanded up to 200- to 1000-fold in 2 to 3 weeks of culture.[4] The expanded CIK cells are heterogeneous lymphocytes with a mixed cell phenotype consisting of a large portion of T cells and small portions of CD3^+^/CD56^+^ natural killer T (NKT) cells and CD3^-^/CD56^+^ NK cells.[6] Although not a major population, CD3^+^/CD56^+^ NKT cells are the key subset of the CIK cells that possess cytolytic activity.[7] These cells show marked up-regulation of the natural killer cell receptor NKG2D (CD314) and can lyse target cancer cells in a major histocompatibility complex (MHC)-unrestricted fashion.[4] Adoptive immunotherapy with CIK cells has been clinically tested for the treatment of solid tumors and exhibit a high safety profile, although further clinical trials are required to verify improvements in disease-free and overall survival.[4,8]

The majority of T lymphocytes in peripheral blood are alpha beta (αβ) T cells with TCRs composed of alpha and beta chains. Only a small subset of T lymphocytes express the gamma and delta heterodimer of TCR chains. γδ TCRs recognize a set of cancer-associated antigens, including phosphoantigens produced during metabolic dysregulation that commonly occurs in tumor cells, specific ligands in the context of antigen presenting CD1 family members, and cell stress markers. As such, γδ T cells have been considered to play important roles in immune surveillance against tumors.[9] Unlike αβ TCRs, γδ TCRs recognize cancer-associated antigens in an HLA-independent manner, thus γδ T cells offer attractive possibilities to be developed as “off-the-shelf” therapeutics. More importantly for cancer treatment, γδ T cells are capable of infiltrating a range of human malignancies, a capacity required for them to interact with and kill cancer cells. These malignancies include renal, bladder, ovarian, colorectal, breast and nasopharyngeal carcinomas.[10] Vγ9Vδ2 T cells, a major subset of human peripheral blood γδ T cells that can be expanded by stimulation with an FDA-approved, commercially available bisphosphonate drug Zoledronic acid (Zometa), have been tested in several early-phase clinical trials of adoptive immunotherapy for cancer with some positive clinical outcomes.[11–13]

While adoptive transfer of different cellular components of the immune system individually for cancer treatment has been widely tested in clinical trials, co-expansion and co-injection of multiple immune cell subsets together to attack different targets in a tumor simultaneously have not been well explored. This combination immunotherapy approach could be attractive given that it might be able to widen the scope of immune responsiveness by engaging a greater number of distinct antigens and provide synergistic activity against cancer. In this aspect, the unique and essential contributions of γδ T cells to many types of immune response have been the subject of extensive research. The crosstalk between γδ T cells and other immune cells has been demonstrated to play an important central role in defending the host against a broad range of stresses.[14]

Based on these clinical and *in vitro* findings, a CAR-based cancer immunotherapy using the combination of CIK and γδ T cells has been proposed. Hence, in the current study, we describe a method for co-expansion of CIK cells and Vγ9Vδ2 T cells, named as CIKZ cells. This method employs a K562 feeder cell-based immune cell expansion protocol that utilizes Zometa, IFN-γ, IL-2 and anti-CD3 antibody together to stimulate peripheral blood mononuclear cells (PBMCs). The antitumor cytotoxicity of the expanded CIKZ cells was observed to be well preserved. We further demonstrated that electroporation with mRNA for anti-CD19 CAR can significantly enhance the anti-Burkitt lymphoma activity of CIKZ cells.

## Materials and Methods

### Ethics statement

The use of fresh buffy coats of healthy donors for human PBMC isolation was approved by the institutional review board of National University of Singapore (NUS-IRB Reference Code B-14-133E) based on the fact that the research uses only anonymous buff coats/apheresis ring belt from the National University Hospital, Department of Laboratory Medicine Blood Transfusion Service.

All handling and care of animals was performed according to the guidelines for the Care and Use of Animals for Scientific Purposes issued by the National Advisory Committee for Laboratory Animal Research, Singapore. The animal study protocol was reviewed and approved by Institutional Animal Care and Use Committee (IACUC), the Biological Resource Centre, the Agency for Science, Technology and Research (A*STAR), Singapore (Permit Number: BRC IACUC 110612).

### Peripheral blood mononuclear cells (PBMCs) and cell lines

Human PBMCs were isolated from fresh buffy coat of healthy donors by density gradient centrifugation using Ficoll-Paque (GE Healthcare, Milwaukee, WI). Human Burkitt lymphoma cell lines Raji (ATCC, Manassas, VA) and Daudi (Sigma-Aldrich, Milano, Italy) and B-cell leukemia cell lines SUP-B15 and Reh (ATCC) were cultured in complete medium RPMI-1640 supplemented with 10% FBS (Hyclone, Logan, UT). Human myelogenous leukemia cell line K562 (ATCC) was cultured in IMDM (Lonza Biotech, Basel, Switzerland) supplemented with 10% FBS. Human primary colon cancer cell line pCRC7 (obtained from a patient’s tumor biopsy, National Cancer Center of Singapore, Singapore), human pharyngeal carcinoma cell line Detrioit562 (ATCC), and human NSCLC cell line H292 (ATCC) were cultured in DMEM supplemented with 10% FBS.

K562 cells were also genetically engineered for stable expression of EGFP, CD86, CD64, and 4-1BBL and used as feeder cells for T cell expansion. The gene encoding sequences for CD64 (FcγRI, GenBank accession no. BC032634), CD86 (B7-2, GenBank accession no. NM_175862) and CD137L (4-1BBL, GenBank accession no. NM_003811) were PCR amplified from a PBMC cDNA library and subcloned into pFastBac1-CMV-EGFP vector to generate pFastBac1-CMV-aAPC3-PuroEGFP. K562 cells were transfected with the vector and selected with 1 μg/ml puromycin (Life Technologies, Carlsbad, CA). A single-cell clone was selected after flow cytometry analysis to confirm CD64, CD137L and CD86 expression. These engineered clonal K562 cells, renamed as K562a, were cultured in IMDM supplemented with 10% FBS and 0.5 μg/ml puromycin to maintain transgene expression and were used for the following cell expansion experiments.

### Immune cell expansion

PBMCs were seeded into 24 well plate at a density of 2×10^6^ cells/ml in AIM-V (Life Technologies) supplemented with 5% human AB serum (Valley Biomedical, Winchester, VA). For the co-expansion of CIK cells and Vγ9Vδ2 T cells (CIKZ method), PBMCs were stimulated by adding 1000 IU/ml IFNγ (PeproTech) and 5 μM Zometa (Sigma Aldrich, St. Louis, Missouri) on day 0, followed by adding 50 ng/ml anti-CD3 antibody (OKT3 clone, eBioscience, San Diego, CA) and 300 IU/mL human recombinant IL-2 on day 1. PBMCs were also stimulated via three other methods: the CIK method by adding 1000 IU/ml IFNγ on day 0, 50 ng/ml OKT3 antibody and 300 IU/mL IL-2 on day 1; the Zometa method with 5 μM Zometa and 300 IU/ml IL-2 on day 0; and the anti-CD3/CD28-dynabead method (Life Technologies). The culture medium was refreshed every 2 to 3 days.

After the cells were activated and expanded for 7 days, they were subjected to a K562a feeder cell co-culture protocol for numerical expansion in the media described above. K562a cells were gamma irradiated at 100 Gy or treated with mitomycin C (20 μg/mL, 37°C for 1 hour), and mixed with immune cells at a 200:1 ratio in T75 or T175 culture flask, followed by adding 5 μM Zometa, 50 ng/ml anti-CD3 antibody (OKT3 clone) and 300 IU/mL IL-2 on day 1 and 300 IU/mL IL-2 thereafter every 2 to 3 days. After 14 days, the co-culture was stopped and phenotypes of expanded immune cells were determined by flow cytometry.

### Cell phenotyping by flow cytometry

Multicolor flow cytometry gating strategy was applied to analyze various cell populations simultaneously. Expanded cells were co-stained with 6 different antibodies and analyzed with LSRII flow cytometer (BD Bioscience, San Jose, CA). Briefly, cells were re-suspended in 100 μl MACS buffer (Miltenyi Biotec, Auburn, CA) and stained with antibody cocktail in the dark at 4°C for 15 minutes. Washing twice with MACs buffer was performed before and after cell staining. The antibody cocktail contains: FITC-conjugated anti-Vδ2 TCR (Biolegend, San Jose, CA), APC-conjugated anti-CD3 (Biolegend), PE conjugated anti-CD4 (BD), PE-CyC7-conjugated anti-CD56, APC-CyC7-conjugated anti-CD16, V450-conjugated anti-CD8a Beckman Coulter (Fullerton, CA). The antibody cocktail used for Treg analysis contains CD4-PE, CD25-APC (BD Bioscience, San Jose, CA), and CD127-FITC (eBioscience) to detect CD4+CD25+CD127- Treg population. The gating strategies used in the current study are included in S1 and S2 Figs. Cell populations were also analyzed with one- or two-color flow cytometry in Accuri C6 flow cytometer (BD). FITC-anti-Vδ2 TCR, APC-anti-CD3, and PE-anti-CD3, and APC-anti-CD56 (Biolegend) were used.

### mRNA CAR preparation

The scFv DNA sequences used for anti-CD19, anti-CEA, and anti HER2 CARs are from Genbank ID: HM852952 (FMC63), Shirasu et al [15], and Carter et al [16], respectively. DNA fragments from the above scFv sequences to CD3zeta were synthesized by Integrated DNA Technologies Pte. Ltd (Singapore) with two digestion sites Sph I and Sal I being included to the 5’ and 3’ ends of the sequence. To construct a CAR expression vector, the synthesized CAR sequence and pFastBac plasmid (Invitrogen) were digested with Sph I and Sal I (New England Biolabs) and ligated together. All the anti-CD19 CAR constructs contained the same scFv fragment, CD8 transmembrane domain and CD3 zeta intracellular domain, but carried different intracellular co-stimulatory domains. The CARs may contain only one co-stimulatory domain CD27 or CD28 (anti-CD19/27 CAR or anti-CD19/28 CAR), or two co-stimulatory domains (anti-CD19/CD28-CD27, or anti-CD19/ CD28-41BB). The membrane-bound GFP (mGFP) CAR was produced by replacing the scFv fragment of anti-CD19/CD28-41BB CAR with the EGFP coding sequence. The anti-CEA and anti-HER2 CARs are the 2^nd^ generation CARs containing the intracellular co-stimulatory domain of CD28. CAR expression was under control of the CMV and T7 promoters. In order to improve mRNA stability upon cell transfection, the CAR expression cassettes were constructed with α-globin 3’ untranslated region (UTR). For the *in vitro* mRNA synthesis, transcription templates were amplified by PCR with a pair of primers designed to amplify the sequence from the CMV promoter to α-globin 3’ UTR. The downstream primer contains a synthesized 150-nt poly(A) tail (Integrated DNA Technologies, Coralville, IA). The PCR products were purified with phenol-chloroform before RNA synthesis with the mMessage mMachine T7 kit (Invitrogen). RNA pellets were re-suspended in RNase-free water and frozen at –80°C for storage.

### Electroporation of mRNA CARs and CAR expression analysis

Expanded CIKZ cells were electroporated using the NEPA21 machine (Nepa Gene Co., Ltd). Briefly, 10~50 x 10^6^ CIKZ cells were washed with PBS and re-suspended in 100 μl of Primary Cell Nucleofector P3 buffer (Lonza, Basel, Switzerland). Before transferring the cell solution into electroporation cuvettes (Lonza), mRNA (10 μg) was added to the cell mixture in P3 buffer. For the mock control, CIKZ cells were electroporated without mRNA addition. The electroporation condition for CIKZ cells was optimized using 250V, 4ms and 1 pulse. Cell culture medium was added into the cuvettes immediately after electroporation, and the transfected cells were cultured overnight for recovery.

To examine CAR expression, the electroporated CIKZ cells were re-suspended in MACs buffer (Miltenyi Biotech, Germany), and incubated with goat IgG (Invitrogen) for 15 minutes to block Fc receptors. Cells were washed and incubated with either biotin-labeled goat anti-mouse-F(ab)2 antibody or biotin-labeled normal goat IgG antibody (Jackson Immunoresearch, West Grove, PA) for 25 minutes. Cells were then washed and stained with PE-labeled streptavidin (BD) and APC-conjugated anti-CD3 (eBiocience) for another 15 minutes. After a final wash, cells were analyzed with Accuri C6 flow cytometer (BD). Isotype control-stained cells were used for population gating.

The electroporated cells were also harvested for Western blot analysis to examine CAR expression. For that, the cells were lysed in radioimmunoprecipitation assay buffer (RIPA) (Santa Cruz Biotechnology) supplemented with proteinase inhibitor (Roche Diagnostics, Pleasanton, CA) and phenylmethyl sulfonyl fluoride (Sigma). The lysis solution was centrifuged, and the protein concentration of the supernatant was measured by Nanodrop^™^ 8000 Spectrophotometer (Eppendorf). Protein was denatured in NuPAGE^®^ LDS loading buffer (Invitrogen) by boiling at 95°C for 5 minutes. Protein samples (60 μg) were separated in 10% acrylamide gradient gel and transferred to polyvinylidene fluoride (PVDF) membrane (Bio-Rad, Hercules, CA) by Trans-Blot SD Semi Dry Transfer Cell system (Bio-Rad). The PVDF membrane was blocked with 5% non-fat milk solution for 2 hours, then incubated with rabbit anti-human anti-CD3 zeta antibody (1:1000 diluted, Sigma) or mouse anti-human anti-β-actin antibody (1:1000 diluted, Santa cruze, CA) overnight at 4°C. The washed membrane was incubated with goat anti-rabbit or goat anti-mouse secondary antibody (1:5000 diluted, Santa Cruze) for 1 hour at room temperature. The membrane was washed and developed with western blotting substrate (Thermo Scientific) and visualized with the ECL machine (Fujifilm, Tokyo, Japan).

### IFNγ ELISPOT assay

IFNγ secretion triggered by tumor antigen specific recognition of anti-CD19 mRNA CARs was determined by IFNγ ELISPOT assay. According to the protocol of IFNγ ELISPOT kit (Mabtech, Nacka Strand, Sweden), the pre-coated ELISPOT plate was washed with PBS and conditioned by blocking medium (AIM-V + 10% FBS) at room temperature for 1 hour. After removing the conditioned medium, effector cells (5 x 10^4^ cells/well) and target cells (5 x 10^3^ cells/well) were seeded into the plate for overnight incubation. The ELISPOT plate was emptied and probed with anti-IFNγ primary antibody and HRP-labeled secondary antibody (100 μl/well) sequentially, for 2-hour and 1-hour incubation at room temperature respectively. After final washing, 100 μl/well of TMB substrate was added into the plate for spots development. The plate was air dried and analyzed by an ELISPOT scanner (CTL, Ltd., of. Cleveland, OH).

### Cytotoxicity assay

The cytolytic activity of expanded CIKZ cells was examined with the DELFIA EuTDA Cytotoxicity Reagents kit (PerkinElmer, Waltham, MA). Target cells were labeled with fluorescence enhancing ligand (BATDA) solution at 37°C for 15 minutes. After extensive wash, cell pellets were re-suspended and the density was adjusted to 5 x 10^4^ cells/ml and mixed with effector cells. The prepared effector and target cells were seeded into 96-well U-bottom plates (Corning, Corning, NY). The effector to target (E:T) ratios used ranged from 40:1 to 0.5:1. Control groups were set up to measure spontaneous release (only target cells added), maximum release (target cells added with 10 μl lysis buffer), and medium background (no cell added). After co-incubation at 37°C for 2 ~ 3 hours, culture plates were centrifuged and 20 μL of supernatant was carefully transferred into flat bottom plates and 200 μL Europium solution was added. The mixed solution was shaken at 250 rpm for 15 minutes at room temperature before reading values with the Victor3 plate reader (Perkin Elmer). Killing efficacy was calculated by using the following formula:

$$\%\text{ Specific release }=\frac{\text{Experimental release }\left(\text{counts}\right)-\text{Spontaneous release (counts)}}{\text{Maximum release }\left(\text{counts}\right)-\text{Spontaneous release (counts)}}\times 100$$

### Animal experiment

NOD-SCID-γc-/-(NSG) mice were bred in house under the approved Institutional Animal Care and Use Committee (IACUC) protocol. To test the *in vivo* anti-tumor activity of CIKZ cells against Raji cells, 10 days after intraperitoneal (i.p.) injection of 5 x 10^5^ Raji cells, tumor-bearing mice were randomly divided into two groups, 10 mice per group, with one group being treated with CIKZ cells and the other with PBS. Ten million CIKZ cells in 100 μl of PBS or the same volume of PBS without cells were i.p. injected into mice once a week for three weeks. Thirty days after Raji cell inoculation, mice were sacrificed through cervical dislocation under sodium pentobarbital anesthesia, and all efforts were made to minimize suffering. Necropsy was performed and tumors found within the i.p. region of the mice were collected. The volume of tumors was determined by measurement of length (L) and width (W) using a digital vernier caliper (Mitutoyo Corp, Kawasaki, Japan) and calculated using the formula: Tumor volume = (L × W × W × 0.5) cm^3^.

To test the *in vivo* anti-Raji activity of CAR-modified CIKZ cells, three days after subcutaneous (s.c.) injection of 5 x 10^5^ Raji cells in the right flank, tumor-bearing mice were randomly divided into three groups, 10 mice per group, and treated with mRNA CAR-modified CIKZ cells or PBS. The mice received 3 i.p. injections, once a week for 3 weeks starting from day 4. Ten million modified CIKZ cells in 100 μl of PBS, or the same volume of PBS without cells, were used for each injection. Behavior and survival of the mice were monitored accordingly. Tumor volume was measured according to the formula described above. Mice were sacrificed when tumor size reached 2 cm^3^ through cervical dislocation under sodium pentobarbital anesthesia, and all efforts were made to minimize suffering. The survival curve was established based on the endpoint of 2 cm^3^ tumor size.

## Results

### T-cell expansion with inactivated K562a cells and characterization of expanded T-cell subpopulations

Different phosphoantigens have been used to expand γδT cells,[17–20] hence we first optimized the condition for phosphoantigen treatment in order to achieve the efficient amplification of a large population of Vγ9Vδ2 T cells. After comparing isopentenyl pyrophosphate (IPP) and Zometa for their potency in Vγ9Vδ2 T-cell expansion, we observed that 5 μM Zometa displayed a relatively high efficiency in activating and expanding Vγ9Vδ2 T cells– 600 fold expansion within 10 days and 48% Vγ9Vδ2 T cells in the expanded cell population (data not shown).

K562 feeder cells are commonly used for αβ T-cell expansion. However, their potential for CIK and γδ T cell expansion is not well established. To develop an efficient method capable of expanding CIK and Vγ9Vδ2 T cells, we constructed genetically engineered K562 cells with surface expression of CD64, CD86 and 4-1BBL. After gene transfer and puromycin selection of the modified cells, they were sorted using an antibody against CD64 and seeded as single cells into 96-well plates that were pre-seeded with gamma-irradiated wild-type K562 cells (5 cells per well) to promote the proliferation of the engineered K562. Several single-cell clones were selected in this way. Flow cytometry analysis of CD64, CD86 and 4-1BBL expression in cells from the selected clone K562a demonstrated that 82.5%, 99.4% and 99.6% of cells express CD64, CD86 and 4-1BBL, respectively (data not shown).

**Fig 1:**
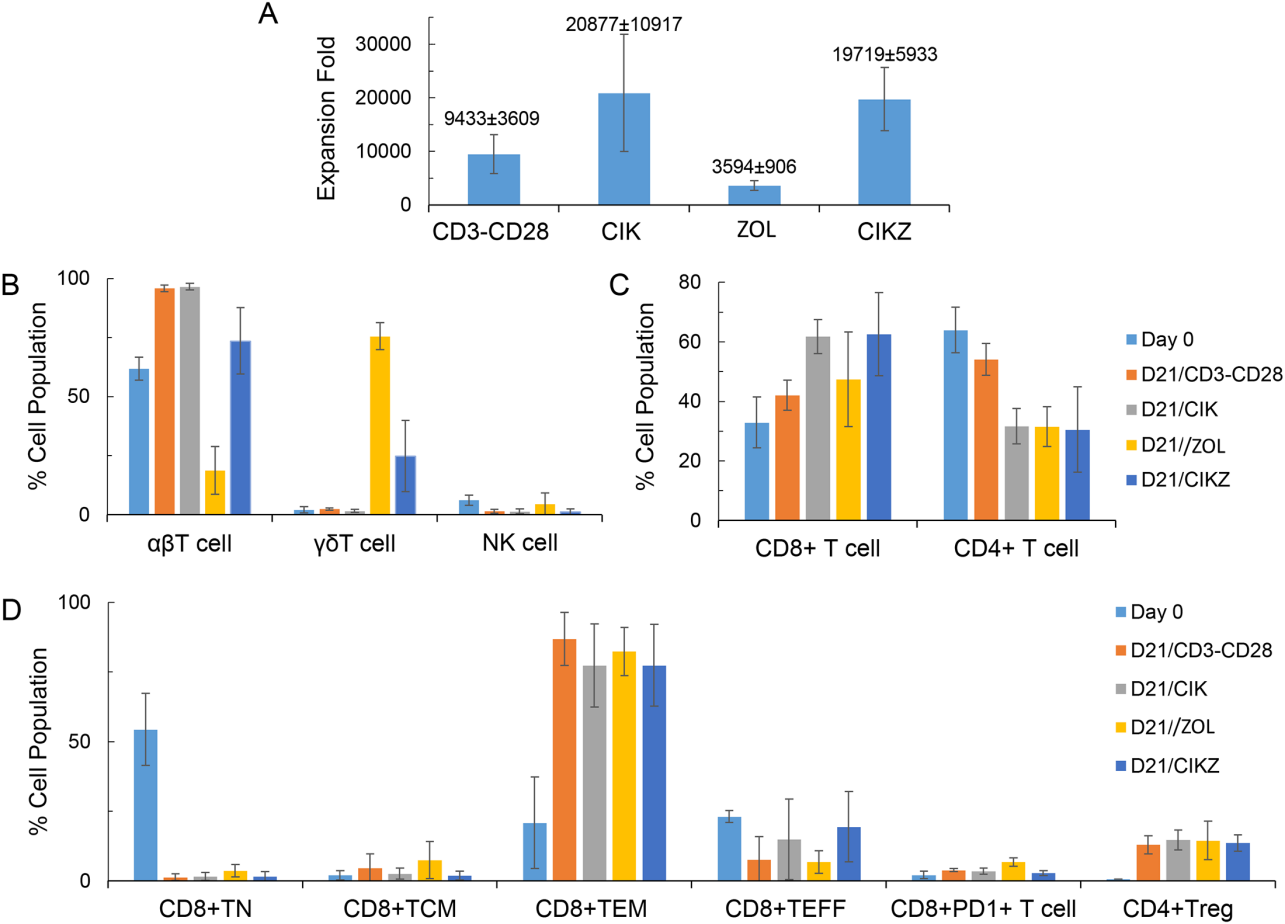
Cell expansion with gamma-irradiated K562a cells and characterization of expanded cells. (A) Expansion fold based on total lymphocyte count after treatment with CD3/CD28 beads, CIK, Zometa (ZOL) or CIKZ methods. PBMCs (2 x 10^6^ per well in a 6-well plate) were first treated with the four methods for 7 days, then 1 x 10^5^ treated cells were mixed with K562a cells at a 1:200 ratio in a T75 flask and expanded for another 14 days. Fold expansion of total cells was determined on days 7 and 21. Data represent the calculated total cell expansion folds based on the actual expansion rates of total cells from Day 0 to Day 7 and Day 7 to Day 21. (B) Frequencies of αβ T, γδ T and NK cells on days 0 and 21. (C) Frequencies of CD8^+^ and CD4^+^ T cells on days 0 and 21. (D) Phenotypic analysis of CD8^+^ and CD4^+^ T cells. TN, TCM, TEM and TEFF: Naïve, central-memory, effector-memory, and effector CD8^+^ T cells, respectively. PD1: Immune exhaustion marker. Treg: CD4^+^ regulatory T cells. Data are displayed as Mean ± SD of total cell expansion fold at the end of the 21-day culture from four donors.

After optimization of different conditions, we developed a standard CIK and Vγ9Vδ2 T cell co-expansion (CIKZ) protocol that consists of two steps. The first step involves stimulating the PBMCs by adding 1000 IU/ml IFNγ and 5 μM Zometa on day 0, followed by adding 50 ng/ml OKT3 antibody and 300 IU/mL human recombinant IL-2 on day 1 for 7 days. The second step involves co-culturing the treated cells with inactivated K562a cells at 1:200 ratio, together with 5 μM Zometa, 50 ng/ml OKT3 antibody and 300 IU/ml IL-2, for 14 days. K562a cells were inactivated with either gamma-irradiation or mitomycin C treatment before use. Applying this CIKZ method in a representative study using PBMCs from 4 donors, a total cell expansion of 19719 ± 5933 fold was achieved at the end of the 21-day culture (Fig 1A). As shown in Fig 1A, this level of total cell expansion was close to that generated by the CIK method without Zometa (20877 ± 10917 fold) and was 2 and 5.5 times greater than that provided by the anti-CD3/CD28-dynabeads (9433 ± 3609 fold) and Zometa (3594 ± 906 fold) methods, respectively. We then analyzed the composition of the *ex vivo* expanded cells after 21 days of expansion. αβ T-cell population (CD3^+^Vδ2^-^) was amplified from ~60% in PBMCs on day 0 to over 90% with the anti-CD3/CD28-beads and CIK methods and ~74% with the CIKZ method (Fig 1B). A decrease in the proportion of αβ T cells to ~20% was observed for the Zometa method. On the other hand, the population of γδ T cells (CD3^+^Vδ2^+^) was expanded from ~1% in PBMCs to over 70% with the Zometa method and over 20% with the CIKZ method; it remained unchanged after expansion with either the anti-CD3/CD28 beads or the CIK method. Also shown in Fig 1B, there was no obvious expansion of CD3^-^CD56^+^ NK cells across the four methods. Among the CD3^+^ αβ T cells, the proportion of CD8^+^ T cells increased after expansion with each of the four methods over 21 days; especially with the CIK and CIKZ methods, the CD8^+^ T-cell population increased from the initial 30% to 60% (Fig 1C). In contrast, CD4^+^ T cells decreased from the starting 60% to about 30% after cell stimulation with the CIK, Zometa or CIKZ method. For all four methods, over 80% of expanded CD8^+^ T cells were effector memory T cells (CD45RA^-^CCRA^-^) and less than 7% expressed the exhausted T-cell marker PD1 (i.e. PD1^+^CD8^+^) (Fig 1D). However, we observed an increase in CD4^+^ Treg-cell frequency from less than 1% in the starting population to approximately 14% after expansion for 21 days across all four methods.

### Expansion of CD3^+^/CD56^+^ NKT subset of CIK cells with tumor-killing function

**Fig 2:**
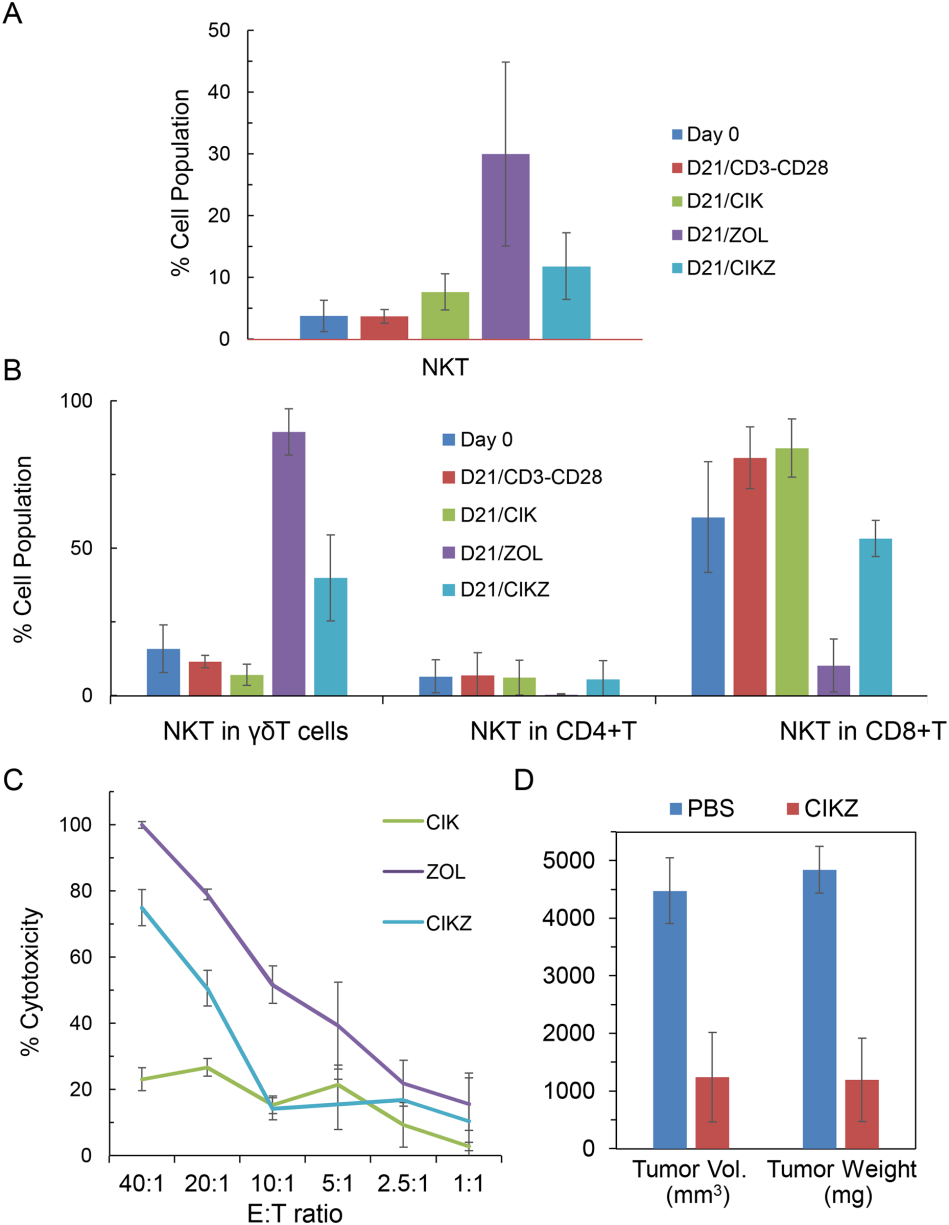
Phenotyping of NKT cell populations and tumor-targeting efficiencies of expanded CIKZ cells. (A) Frequencies of the total CD3^+^CD56^+^ NKT cells were examined by flow cytometry before and after cell expansion with CD3/CD28 beads, CIK, Zometa (ZOL) or CIKZ methods. (B) The detailed phenotypic analysis of CD3^+^CD56^+^ T cells after gating with γδ T, CD4^+^ T and CD8^+^ T cell markers. (C) Cytolytic activities of the cells expanded with CIK, ZOL and CIKZ methods against Raji human malignant Burkitt lymphoma cell line. Effector and target cells were co-cultured at the indicated cell ratios for 3 hours before measuring cytolytic activities. (D) *In vivo* effects of CIKZ cells on Raji tumor growth. Tumor volumes and weights of in Raji tumor-bearing mice sacrificed 30 days after i.p. tumor inoculation. P value of tumor volume was found to be p < 0.05 while P value of tumor weight was p < 0.01 between PBS- and CIKZ-treated mice.

Since CD3^+^/CD56^+^ NKT cells are the key subset of the CIK cells responsible for cytotoxicity, we analyzed the NKT-cell frequency in the resultant populations after 21 days of expansion. While there was no change after the 21-day expansion with the anti-CD3/CD28-dynabeads method, we observed increases in NKT-cell frequency with the CIK, Zometa and CIKZ methods from less than 4% at day 0 to 8%, 30% and 12%, respectively, at day 21 (Fig 2A). Since both γδ T and αβ T cells can express CD56,[19] we gated the CD3^+^/CD56^+^ cells from the γδ T, CD4^+^ αβ T and CD8^+^ αβ T cells separately to analyze the various NKT cell subpopulations. As shown in Fig 2B, we observed that the proportion of Vδ2^+^ NKT cells increased mainly after the use of Zometa—from 16% on day 0 to 90% and 40% on day 21 with the Zometa and CIKZ methods respectively. There were no significant changes in the population of CD4^+^ NKT cells over 21 days with the four methods but the CD8+ NKT-cell population increased by approximately 20% when cells were stimulated by the anti-CD3/CD28-dynabeads and CIK methods (Fig 2B). Overall, the results indicate that expansion with stimulation by the Zometa method yielded the highest proportion of γδ T cells and CD3^+^/CD56^+^ NKT cells among the four methods, with the CIKZ method the next highest. However, the significantly higher fold expansion achieved by the CIKZ method makes it the most efficient for the co-expansion of γδ T cells and CIK cells. Based on the total cell expansion fold and γδ T cell percentage before and after expansion, γδ T cell expansion folds by the CIKZ method and Zometa method are 394,380 and 251,580 respectively, demonstrating again the superiority of the CIKZ method in terms of ex vivo expansion of γδ T cells.

We further examined the cytolytic activity of the expanded cells stimulated with the CIK, Zometa and CIKZ methods using Raji cells as target cells. Raji cells were relatively resistant to the cytolytic action of the cells expanded with the CIK method but were effectively killed by the cells expanded with the Zometa and CIKZ methods (Fig 2C). The *in vivo* tumor-killing effects provided by CIKZ cells was confirmed in a Raji tumor model established by i.p. injection of Raji cells into NSG mice (Fig 2D). The treatment with CIKZ cells reduced the volume and weight of the Raji-cell tumors by about 70%.

### Modification of cells expanded with the CIKZ method with various anti-CD19 RNA CARs

**Fig 3:**
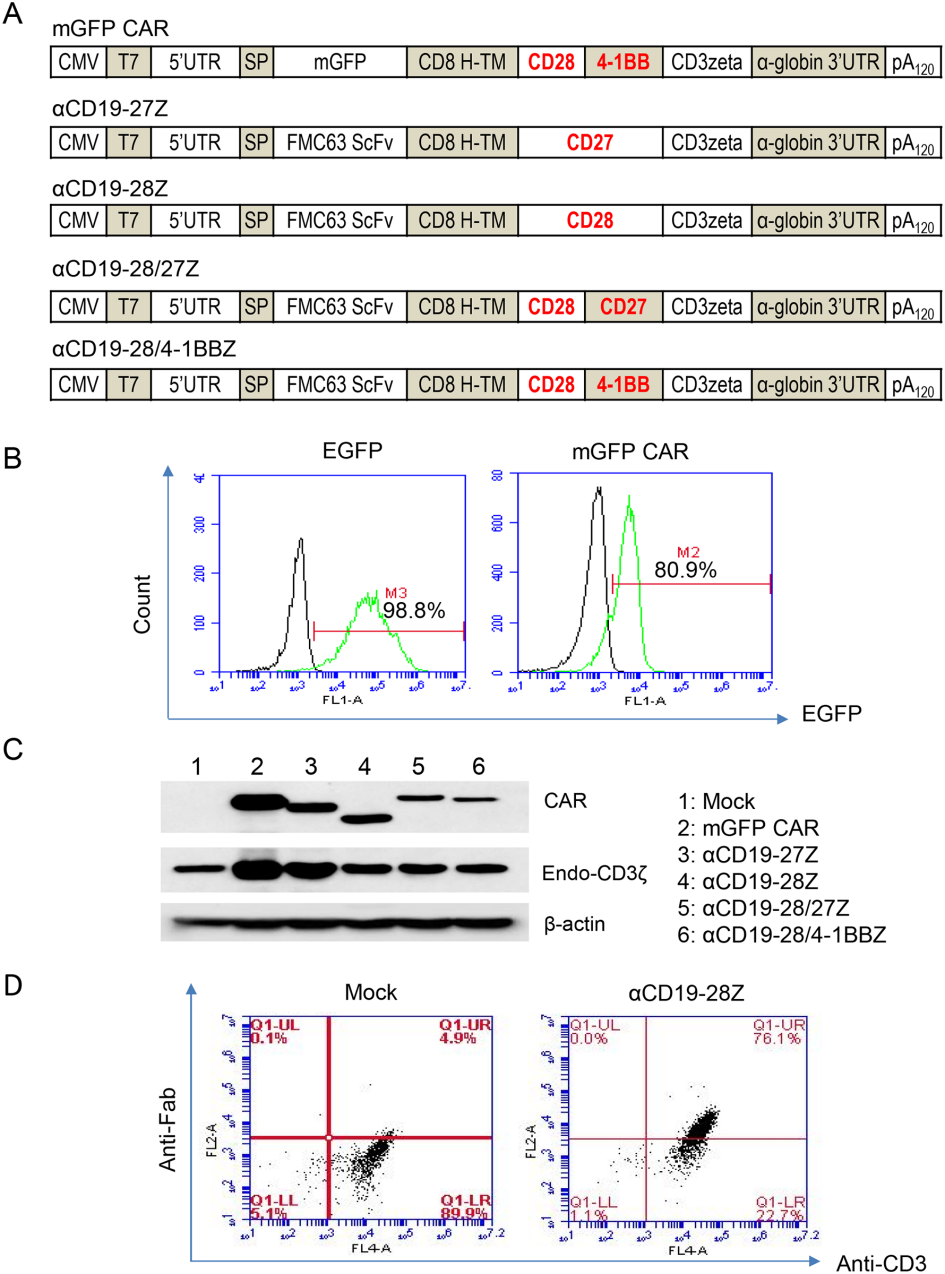
Anti-CD19 CAR design and expression in cells expanded with the CIKZ method. (A) Schematic diagrams of mRNA CAR constructs used in this study. The anti-CD19 CARs listed share the same scFv fragment but carry different co-stimulatory domains. mGFP CAR was constructed by replacing the scFv fragment of anti-CD19 CAR(CD28-41BB) with the EGFP codon sequence and used as the CAR control. (B) The electroporation efficiency in CIKZ cells. The cells were transfected with normal EGFP mRNA for cytoplasm expression and mGFP CAR mRNA for membrane expression and GFP expression was examined by flow cytometry. (C) RNA CAR expression in CIKZ cells was analyzed by Western blot through the detection of the exogenous CD3 zeta chain-containing chimeric protein (approximately 60 kD). The endogenous CD3 zeta chain (about 20 kD) could also be recognized by the antibody. β-actin was included as the loading control. (D) CAR expression on the CIKZ cell surface was determined by anti-Fab flow cytometry. Untransfected CIKZ cells were co-stained with PE-conjugated isotype antibody and anti-CD3 APC and used as the gating control.

Equipping immune cells with CAR can enhance their activities towards cancer cells. Since CIKZ cells can be more extensively expanded than γδ T cells alone while still exhibiting a relatively high cytolytic activity, we proceeded to test whether anti-CD19 CAR-redirected CIKZ cells display improved cytotoxicity against CD19-positive cancer cells. We were particularly interested in investigating the effects of new anti-CD19 CAR constructs with the CD27 domain and constructed both the second (with one co-stimulatory domain) and third (with two co-stimulatory domains) generation CARs as illustrated in Fig 3A. We adopted an RNA CAR approach to facilitate fast evaluation of different CAR constructs. RNA electroporation was optimized in CIKZ cells using EGFP mRNA, which provided almost 99% transfection efficiency (Fig 3B). The electroporation of CIKZ cells with mGFP CAR, a CAR control construct with mGFP to replace the anti-CD19 single-chain variable fragment (scFv) in an anti-CD19 CAR construct, gave about 81% transfection efficiency (Fig 3B). Following electroporation of CIKZ cells with RNA CAR, expression of the anti-CD19 CAR constructs was confirmed with Western blotting using an antibody against the CD3 zeta chain (Fig 3C). While only the endogenous CD3 zeta chain was detected in un-transfected CIKZ cells, both the exogenous and endogenous CD3 zeta chains were detected in CIKZ cells transfected with the anti-CD19 CAR constructs. Overall, the expression of the second generation CARs (anti-CD19/CD27 and anti-CD19/CD28 CARs) was higher than that of the third generation CARs (anti-CD19/CD28-27 and anti-CD19/CD28-41BB CARs). As shown in Fig 3D, anti-Fab flow cytometry analysis confirmed that the expression efficiency after the transfection of the anti-CD19-28Z was 71.2% (after subtracting nonspecific fluorescence) in CIKZ cells.

### CIKZ cells modified with various CD19-specific mRNA CARs show different tumor-targeting efficiencies and cytotoxicity

**Fig 4:**
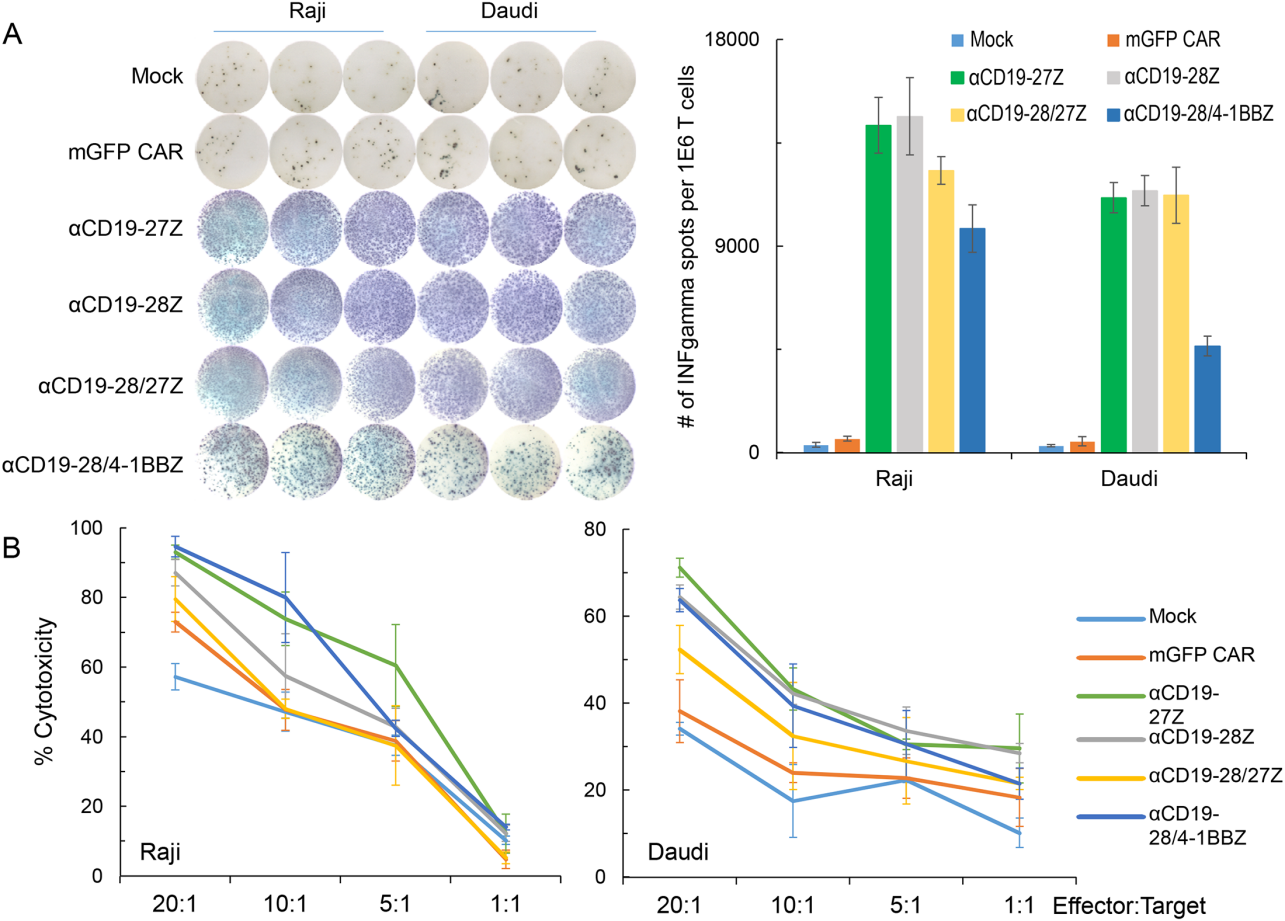
Tumor-targeting efficiencies and cytotoxicity of CIKZ cells modified with various anti-CD19 mRNA CARs. (A) IFNγ secretion triggered by tumor antigen-specific recognition of anti-CD19 mRNA CARs as determined by IFNγ ELISPOT assay. CIKZ cells were electroporated with indicated mRNA CARs and co-cultured with target tumor cells overnight before ELISPOT assay. Mock-transduced T cells served as a negative control. After co-culturing with Raji and Daudi cells. Images from the plate scan of the ELISPOT assay are shown on the left. Mean IFNγ spots per 1 x 10^6^ CIKZ cells ± SD from triplicate cultures is shown on the right. (B) *In vitro* killing efficiencies against Raji and Daudi cells provided CIKZ cells equipped with anti-CD19 mRNA CARs.

To compare the tumor-targeting efficiencies of various CD19-specific mRNA CARs, the same amount of mRNA CARs were transfected into CIKZ cells by electroporation. By using CD19^+^ Raji and Daudi cells as targets, the CD19-specific CAR-mediated CIKZ-cell stimulation and cytokine secretion were determined by IFNγ ELISPOT assay since IFNγ secretion is associated with antitumor activity of T cells for adoptive immunotherapy. The scanned ELISPOT plate in Fig 4A directly showed that there was stronger development of spots with CD19-specific CAR-equipped CIKZ cells than with the control CIKZ cells. Correspondingly, analysis with a spot-counting machine indicated that the number of spots for all CD19-specific CAR-modified CIKZ cells was significantly higher than for mock-CIKZ or mGFP CAR CIKZ cells (P < 0.001). Comparing among the different mRNA CAR-modified CIKZ cells, the IFNγ secretion levels of anti-CD19/CD27 CAR- and anti-CD19/CD28 CAR-equipped CIKZ cells were comparable to each other; the IFNγ release of anti-CD19/CD28-27 CAR-CIKZ cells was relatively lower than the second generation CARs when targeting Raji but was not significantly different when the target was Daudi. Additionally, the IFNγ release of the anti-CD19/CD28-41BB CAR-CIKZ cells was significantly lower than all the other anti-CD19 CAR-modified cells.

To determine the cytotoxicity of CIKZ cells after genetic modification with variant CD19-specific CAR mRNAs, an *in vitro* cytotoxicity assay was performed using the Delfia cytotoxicity kit. The same amount of mRNA for each anti-CD19 CARs was electroporated into CIKZ cells. With Raji cells as the target, the killing efficacy of CIKZ cells post anti-CD19 CAR mRNA modification significantly increased to over 90% from the approximately 60% cytotoxicity observed for the mock and mGFP CAR controls at an effector to target ratio of 20:1 (Fig 4B). Among all the anti-CD19 mRNA CARs, the newly generated anti-CD19/CD28-27 mRNA CAR-modified CIKZ cells exhibited the lowest killing efficacy irrespective of the target cell type as shown in Fig 4B. Although the IFNγ release of the anti-CD19/CD28-27 mRNA CAR-modified cells was comparable to that of the cells modified with second generation mRNA CARs as shown in Fig 4A, the cytotoxic function of CD19/CD28-27 mRNA CAR was found to be relatively weaker (Fig 4B). As for the other three anti-CD19 mRNA CARs, there was no significant difference among them with respect to their killing efficacies when targeting Daudi cells. Hence, the conventional CAR constructs using CD27, CD28, or CD28-41BB as co-stimulatory domains appeared to be superior to the CAR with the CD28-CD27 co-stimulatory domain. It is also noted from Fig 4B that anti-CD19/CD27 CAR- and anti-CD19/CD28-41BB CAR-modified CIKZ cells exhibit relatively higher killing efficacy than anti-CD19/CD28 CAR against Raji cells at higher effector to target ratios (10:1 and 20:1). However, the cytotoxicity of anti-CD19/CD28 CAR-modified CIKZ cells was marginally higher than that of anti-CD19/CD28-41BB CAR and comparable to anti-CD19/CD27 CAR when the target cell was Daudi and at lower effector to target ratios. Based on these results of the *in vitro* cytotoxicity assay, we deduced that the new CAR design, anti-CD19/CD28-27 CAR, was not as effective as conventional CAR designs. We also determined that anti-CD19/CD27 CAR and anti-CD19/CD28 CAR were comparable to each other with respect to cytokine release and cytotoxicity against tumor cells. Additionally, anti-CD19/CD28-41BB CAR-modified cells exhibit good killing efficiency, but cytokine release of the cells was much lower than the second generation CARs.

### CD28 CAR-modified CIKZ cells provide a new platform for adoptive T cell therapy

**Fig 5:**
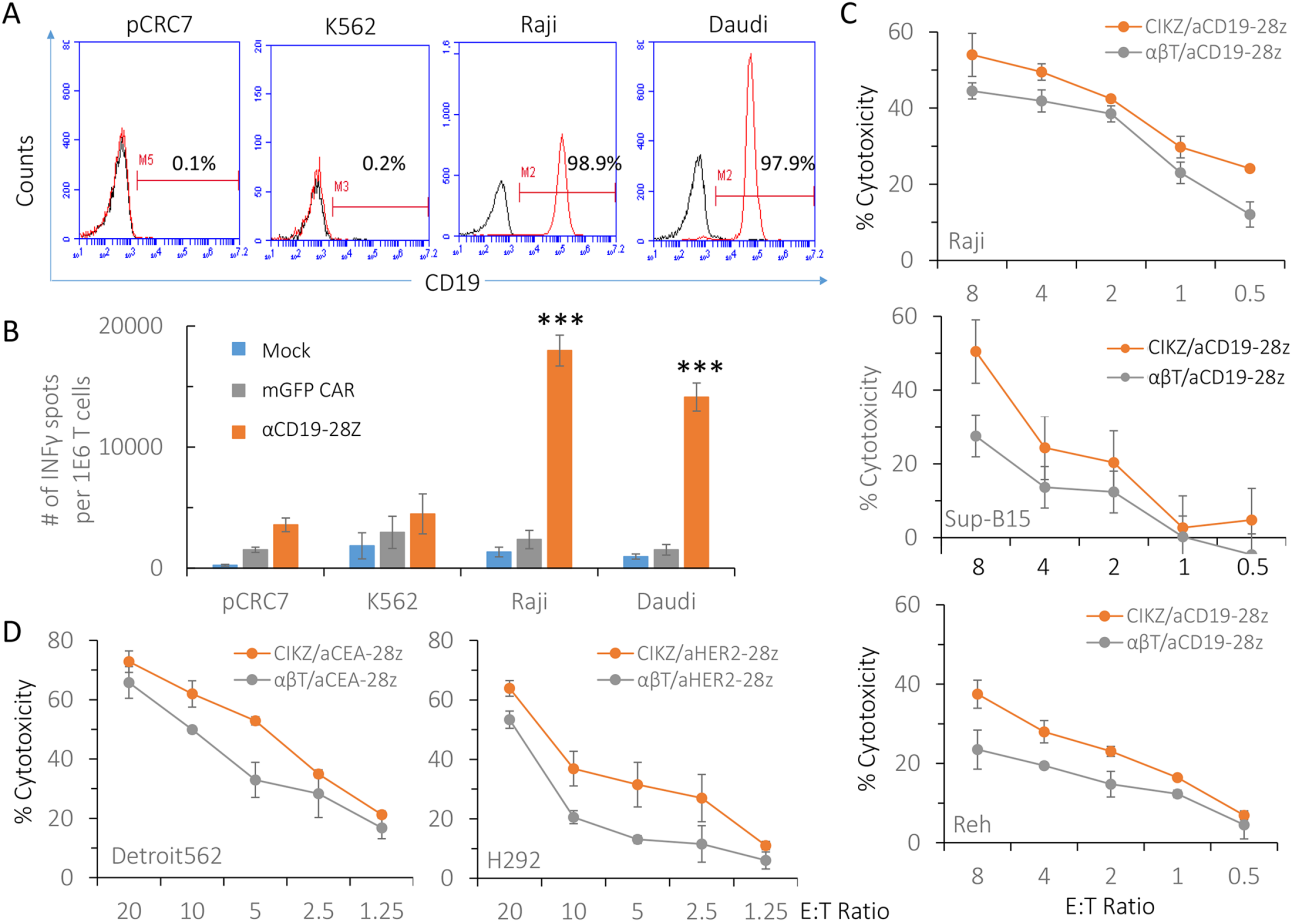
Cytotoxic immune responses induced by CD28 CAR-modified CIKZ cells. (A) and (B) The specificity of anti-CD19/CD28 CAR in targeting CD19-positive tumor cells. Results of flow cytometric analysis of CD19 expression on 4 different types of cancer cells are shown in (A) and the specificity of anti-CD19 CAR is demonstrated in (B), after co-culturing with CD19-negative cells pCRC7 colorectal cancer cells and K562 cells and CD19-positive Raji and Daudi cells. Mean IFNγ spots per 1 x 10^6^ CIKZ cells ± SD from triplicate cultures are shown. (C) Cytolytic effects of CIKZ cells and αβ T cells electroportated with anti-CD19/CD28 CAR mRNA on other CD19-positive B-cell leukemia cell lines Raji, SUP-B15, and Reh at limiting doses. (D) Cytolytic effects of CIKZ cells and αβ T cells electroportated with anti-CEA/CD28 CAR mRNA (Left) and anti-HER2/CD28 CAR mRNA (Right). CEA-positive human pharyngeal carcinoma cell line Detrioit562 and HER2-positive human NSCLC cell line H292 were used as target cells.

Next, we investigated the specificity of the anti-CD19 CARs, focusing on anti-CD19/CD28 CAR against both CD19 positive and negative tumor cells. We first verified by flow cytometry that the colorectal cancer cell line pCRC7 and K562 cells did not express surface CD19 while Raji and Daudi cells were CD19 positive (Fig 5A). Using anti-CD19/CD28 CAR-modified CIKZ cells as effector cells in an IFNγ ELISPOT assay, the number of IFNγ spots that developed was more than 3 times higher, with statistical significance (p < 0.001), when target cells were Raji or Daudi rather than pCRC7 and K562 cells. Meanwhile, IFNγ secretion of mock CIKZ cells and mGFP CAR CIKZ cells were consistently low among the different target cells regardless of CD19-expression on target cell surface (Fig 5B). Based on the result, we understand that the reaction between anti-CD19 CAR-modified CIKZ cells and target cells was tumor antigen-specific. To further investigate cytolytic effects of anti-CD19/CD28 CAR and also evaluate performance differences between CAR-expressing CIKZ cells and αβ T cells, which are conventionally used in CAR T cell therapy, we conducted *in vitro* cytotoxicity assays with anti-CD19/CD28 CAR-modified CIKZ cells and αβ T cells at limiting doses to compare their killing efficacies against three CD19-positive B-cell leukemia cell lines Raji, SUP-B15, and Reh. As shown in Fig 5C, the CAR-modified CIKZ cells exhibited killing efficacy relatively higher than the CAR-modified αβ T cells irrespective of the target cell type. To evaluate whether the potential advantages of CAR-modified CIKZ cells compared to αβ T cells extends beyond the CD19 antigen, we generated two 2^nd^ generation CAR constructs with the cytoplasmic domain of CD28 directed against human carcinoembryonic antigen (CEA) and human epidermal growth factor receptor 2 (HER2), and transfected CIKZ cells and αβ T cells by electroporation of anti-CEA/CD28 CAR mRNA and anti-HER2/CD28 CAR mRNA respectively. With CEA-positive human pharyngeal carcinoma cell line Detrioit562 and HER2-positive human NSCLC cell line H292 as target cells, immune cells modified by both CAR constructs exerted dose-dependent, cytotoxic effects on tumor cells, whereas CAR-modified CIKZ cells displayed killing efficacy relatively higher or comparable to CAR-modified αβ T cells (Fig 5D). Thus, our findings suggest that CAR-modified CIKZ cells exhibit comparable or superior performance to more "conventional" CAR-T cells in mediating *in vitro* tumor cell cytolysis.

### Anti-CD19/CD28 mRNA CAR-modified CIKZ cells show *in vivo* tumor-killing effect in a Raji tumor mouse model

**Fig 6:**
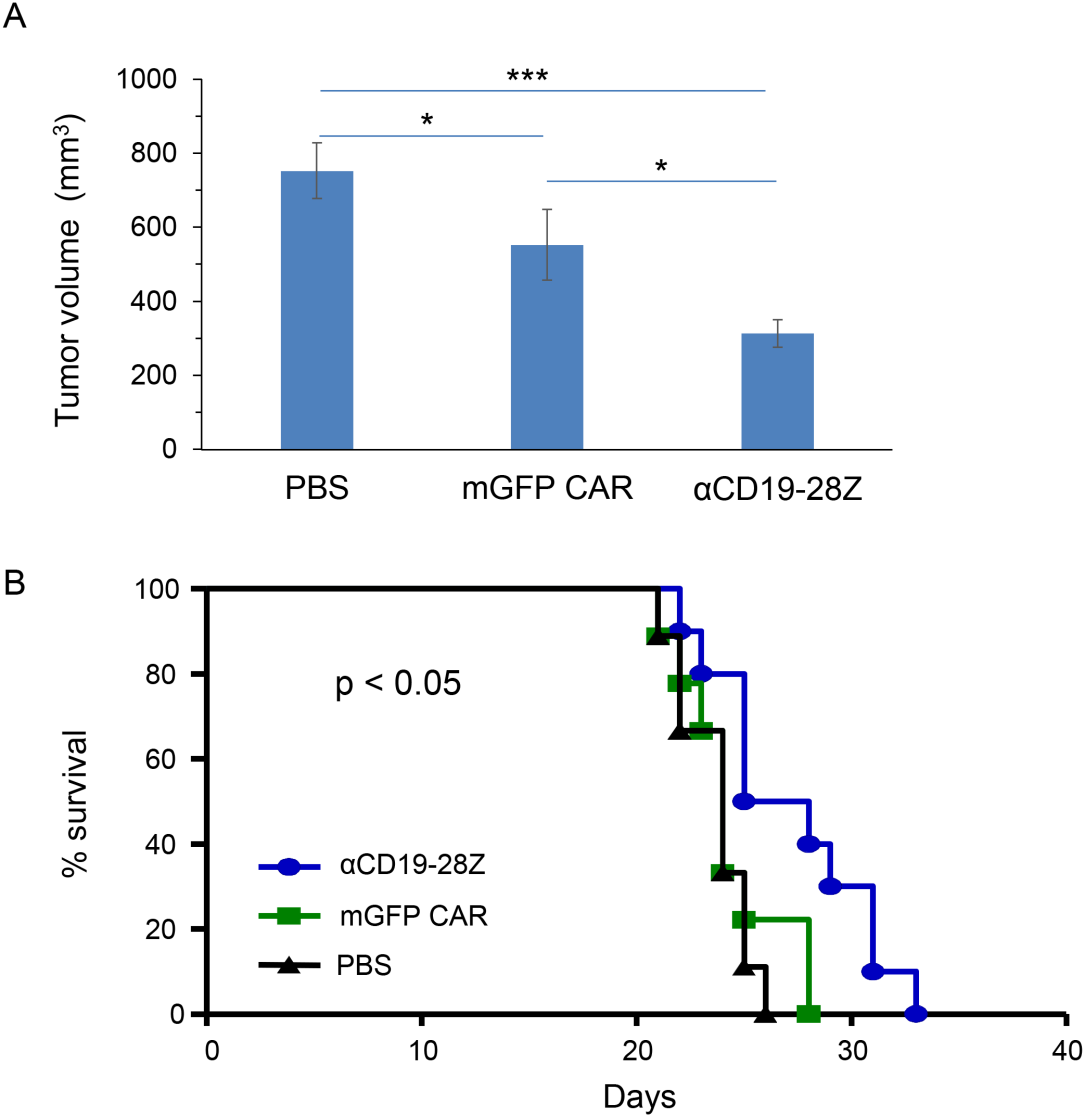
*In vivo* cancer cell-killing by CIKZ cells equipped with anti-CD19 mRNA CARs in a Raji tumor mouse model. (A) Tumor volume measured after 18 days post-Raji injection. p < 0.001 between PBS- and aCD19-28Z-treated mice and p < 0.05 between mGFP CAR- and aCD19-28Z-treated mice. (B) Survival curve. Mice whose tumor size reached 2 cm^3^ were euthanized and deemed “non-survival”. The statistical analysis of survival data was performed using the log-rank test, p < 0.05.

Because anti-CD19/CD28 CAR is more widely used in CAR-T cell therapy, we modified CIKZ cells with anti-CD19/CD28 mRNA CAR to demonstrate the *in vivo* tumor-killing efficiency of mRNA CAR-modified CIKZ cells. A B-cell lymphoma mouse model was established by subcutaneous injection of Raji cells into the right flank of NSG mice. Three days post-Raji injection, the first dose of the CAR-CIKZ cells was i.p. injected into the mice. Another two doses were administered in the following two weeks. Control mice were administered with either the same volume of PBS or the same cell number of mGFP CAR CIKZ cells. We observed that the tumor growth in the effector cell-treated groups was significantly decreased compared with the PBS control group. Based on the measurement of tumor size on day 18 post-Raji injection as shown in Fig 6A, tumor size in the anti-CD19 CAR CIKZ group was significantly smaller than that in the mGFP CAR CIKZ group (p < 0.05) and PBS group (p < 0.001). Although tumors continued to grow over the following 3 days, the size of tumors in the CIKZ cell-treated groups remained significantly smaller than that in the PBS group (data not shown). In addition, the tumor size in the mGFP CAR CIKZ group was also significantly smaller than in the PBS group. Hence, tumor growth was suppressed by three injections of mGFP CAR CIKZ cells or anti-CD19 mRNA CAR-modified CIKZ cells. This finding indicates that CIKZ cells alone are able to effectively inhibit tumor growth and that the tumor-killing efficiency can be further enhanced through mRNA CAR modification. Mice were sacrificed once tumor size reached 2 cm^3^. Survival curve was created based on the date of mouse euthanization. As shown in Fig 6B, date of the last mouse euthanized in each group was day 25, day 28 and day 33 in the PBS, mGFP CAR CIKZ and anti-CD19 CAR CIKZ cell treated groups respectively. The median survival of PBS and mGFP CAR CIKZ cell treated mice was 24 days while anti-CD19 CAR CIKZ cell treated mice was 26.5 days, hence, survival in mice was improved through CAR-modified CIKZ cell injection, with statistical significance (p < 0.05). Even though mGFP CAR CIKZ cells did not significantly increase mice survival, tumor growth was still inhibited, especially in the early state of tumor development (before day 22). This likely suggests that more frequent administration or higher dose of CIKZ cells alone is helpful to control tumor growth, and mRNA CAR modification provides a possible way to further improve the tumor-targeting therapeutic efficiency of CIKZ cells.

## Discussion

Many immune cell expansion methods have been developed to meet the demands for adoptive cell-based cancer immunotherapy. In several protocols to generate CIK cells, patient-isolated PBMCs are stimulated with IFN-γ, IL-2 and anti-CD3 antibody for 3 to 4 weeks.[4,6,21,22,23] The timed addition of IFN-γ at the beginning of the expansion is noted to be critical in activating monocytes present in PBMCs to provide contact-dependent (CD58/LFA-3) and soluble (IL-12) factors that direct them towards acquiring the Th1 phenotype and cytotoxic function of CIK cells.[24] IFN-γ directly suppresses the proliferation of Th2 cells, thus inhibiting a large fraction of CD4^+^ cells and favoring the growth of CD8^+^ and TH1 cells in CIK cultures.[25] The anti-CD3 antibodies provide an initial mitogenic signal, which is sustained by the continuous presence of IL-2. Hundred-fold expansion of total cells is typically observed after 3 to 4 weeks of culture, although expansion rates up to thousand-fold are also reported.[4]

Deniger et al. have published a method describing how to activate and propagate polyclonal gamma delta T cells with broad specificity for malignancies. [26,27] To prepare Vγ9Vδ2 T cells for adoptive cell immunotherapy, patient PBMCs typically isolated via leukapheresis, which yield around 1 x 10^9^ PBMCs, undergo expansion stimulated by a Vγ9Vδ2 ligand to produce Vγ9Vδ2 T cells necessary for multiple injections.[11,18,20] Since Vγ9Vδ2 T cells represent only a small fraction, approximately 0.5–5%, of peripheral blood T cells, extensive expansion of Vγ9Vδ2 T cells is necessary to provide the large number of cells required for many adoptive immunotherapy applications to achieve clinical benefit. IPP, the natural antigen for Vγ9Vδ2 subset of human γδ T cells, has been used for expansion of γδ T cells.[15] More recently, the synthetic analogue of IPP bromohydrin pyrophosphate (BrHPP) and Zometa have been demonstrated to selectively activate human Vγ9Vδ2 T cells in clinical trials and are now widely used for *ex vivo* expansion of Vγ9Vδ2 T cells.[10,11,19] Zometa effectuates an increase intracellular levels of IPP in immune cells by inhibiting farnesyl pyrophosphate synthase (FPPS), an enzyme acting downstream of IPP in the mevalonate pathway.[19] The practical benefits of using Zometa are that it is commercially available and FDA-approved for clinical treatments such as for postmenopausal osteoporosis.

Clinically, it is crucial for adoptive cancer immunotherapy that effective methods are developed for large-scale expansion of patient immune cells (typically up to 5 × 10^10^ cells are required). The expansion methods also need to be rapid enough to make adoptive cell therapy for cancer practical and accessible. For the expansion of CD3^+^ immune cells, this can be achieved using a traditional feeder cell method, called the “rapid expansion protocol” (REP), in which cells are expanded with anti-CD3 antibody, IL-2, and feeder cells such as irradiated allogeneic PBMCs.[28] The Fc-portion of anti-CD3 antibody (e.g. OKT-3) attaches to Fc-γ I receptor (FcγRI) expressed on the feeder-cell PBMCs, whereas its antigen-binding sites interact with CD3 on the immune cell surface. This cell-cell interaction between CD3^+^ immune cells and FcγRI^+^ feeder cells provides co-stimulation on top of anti-CD3-T-cell receptor (TCR) crosslinking.[29] Anti-CD3-TCR crosslinking and the interaction between T cells and PBMCs can synergize to deliver signal-1 and co-stimulatory signal-2, leading to T-cell proliferation without triggering anergy or apoptosis. REP can result in a thousand-fold expansion of T lymphocytes over a 14-day period and has been used extensively for expansion of CD3^+^ immune cells for clinical adoptive transfer studies.[11,30] However, the REP approach presents some logistic and technical limitations that may prevent large multisite randomized clinical trials. For one, it needs large numbers of allogeneic PBMCs collected by large-volume leukapheresis from multiple healthy donors as a 200:1 ratio of PBMC feeder cells to T cells is typically used for clinical propagation.[11] Allogeneic PBMCs collected for REP will need to go through extensive testing to confirm sterility, which could be costly and time-consuming. Moreover, the PBMCs collected from multiple donors might exhibit donor to donor variability in terms of supporting CD3^+^ immune cell expansion.

Recently, artificial antigen-presenting cells (aAPCs) with expression of co-stimulatory molecules on their surface have been demonstrated to support robust *ex vivo* immune cell expansion in a manner commensurate with the numerical expansion achieved by REP.[27,28] Human erythroleukemia cell line K562 is often used as a backbone to generate aAPCs through genetic engineering to introduce exogenous co-stimulatory molecules.[31] K562 cells reportedly express neither endogenous HLA class I and II molecules, thus minimizing the unintended allogeneic responses, nor co-stimulatory molecules such as CD86, CD83, 4-1BBL, OX40L, ICOSL (B7H2, B7RP1), or CD40L.[32] K562 cells also lack the expression of inhibitory molecules, such as PDL1 (B7H1), PDL2 (B7DC), B7H3, and B7H4 (B7X, B7S1).[33] Among three human IgG Fc receptors, K562 endogenously expresses a high level of CD32 but not CD16 or CD64.[31] K562 cells also express adhesion molecules CD54 (ICAM-1) and CD58 (LFA-3), which enhance K562 interactions with T cells and improve T cell stimulation.[32] Furthermore, K562 cells can be easily manipulated for stable expression of transgenes and are confirmed to be safe in clinical trials when used as feeder cells for T cell expansion.[31] K562-based aAPCs have been successfully used to expand polyclonal CD3+ T cells, antigen-specific CD8+ T cells, antigen-specific CD4+ T cells, CAR-transduced T cells, invariant natural killer T (iNKT) cells, and NK cells. [31] However, their application in the expansion of other types of lymphocytes, such as CIK cells and γδ T lymphocytes, are not well established.

As such, the current study reports the development of a method utilizing K562-based aAPCs for large-scale expansion of CIK cells and γδ T cells simultaneously. K562 cells have been genetically engineered to stably express three genes encoding the high affinity Fc receptor CD64 and co-stimulatory molecule ligands CD86 and 4-1BBL, the ligand of the 4-1BB receptor. The 4-1BB receptor is a co-stimulatory molecule preferentially expressed on activated T cells. Engagement of 4-1BB signaling inhibits activation-induced cell death, favorably expanding CD8^+^ T cells over CD4^+^ T cells.[34] 4-1BB signaling also enhances the development of a memory phenotype of CD8^+^ T cells.[35] CD86 is the ligand of the CD28 co-stimulatory receptor that is expressed on both resting and activated T cells. Through CD64 interaction with the Fc-portion of the OKT3 monoclonal antibody against CD3, T lymphocytes will be stimulated by both signal-1 (OKT3-CD3) and the CD86-CD28 and 4-1BBL-4-1BB co-stimulatory signals. The results in this study demonstrate that the use of the engineered K562 aAPCs in our newly developed expansion protocol with Zometa, IFN-γ, IL-2 and anti-CD3 antibody provides a powerful approach for the efficient *ex vivo* bulk expansion of functional CIK cells and Vγ9Vδ2 T cells, surpassing existing γδ T-cell expansion methods in terms of absolute Vγ9Vδ2 cell numbers generated. The antitumor cytotoxicity of the expanded lymphocytes appears to be well preserved and can kill both solid and non-solid tumor cells. Similar to γδ T cells and CIK cells, CIKZ cells exhibit inherent cytotoxicity against tumor cells, such as Daudi and K562 cells. Compared with cells expanded by the standard CIK method, CIKZ cells exhibit greater cytotoxicity (Fig 2C). Although the cytotoxicity of CIKZ cells was lower than that of γδ T cells generated by the Zometa method, the cell expansion of CIKZ cells was much greater than that of γδ T cells. In general, we found that CIKZ cells are able to rapidly expand and maintain inherent cytotoxicity against tumor cells.

While extensive cell expansion over a short period of time and non-MHC-restricted tumor killing are important factors to consider in clinical trials, just as crucial are the safety and specificity of the expanded cells for adoptive cancer immunotherapy. Since CIKZ cells are expanded using the combination of the γδ T and CIK cell generation protocols, we surmise that the immunity of CIKZ cells should be close to γδ T and CIK cell, and the likelihood of graft-versus-host disease (GVHD) caused by transfusion of CIKZ cells is likely to be as low as that for γδ T and CIK cells expanded via the Zometa and CIK methods.[8] In terms of function, our xenograft study revealed that CIKZ cells exhibit tumor inhibition capability *in vivo* (Fig 6).

One aim of this work is to provide the proof of concept that CIKZ cells can be used as an alternative cell source for adoptive CAR cell therapy. With CD19 as the target, various 2nd and 3rd generation CARs have been tested pre-clinically and clinically, which include anti-CD19/CD27, anti-CD19/CD28, anti-CD19/4-1BB, anti-CD19/OX40, and anti-CD19/CD28-4-1BB CARs. We compared cytotoxic immune responses induced by CIKZ cells modified with various mRNA CARs, in view of the possibility that transient CAR-expressing effector cells generated by mRNA CAR transfection can be employed to achieve antitumor effects through multiple infusions and yet control excessive responses caused by on-target/off-tumour toxicity and cytokine storms through discontinuing the infusion of the transfected effector cells. Other studies on CAR T-cell immunotherapy have previously shown that T cells modified by DNA-based gene transfer for long-term CAR expression may exhibit cytotoxicity due to the cytokine storm and cross-toxicity against normal tissue cells.[28,36] In contrast, mRNA-based CAR expression, in which mRNA encoding a CAR molecule is electroporated into cells, provides short-term expression of CAR on the cell surface (lasting a few hours to 1 week post electroporation) and, hence, is feasible to reduce or stop toxicity by ceasing administration of CAR T cells. Moreover, mRNA electroporation is straightforward and low in cost compared with lentivirus- or retrovirus-based CAR T cell production. In addition, GMP-grade mRNA production is robust and easy-to-perform, allowing for large-scale production.[28,37] Based on the mRNA CAR platform, it is possible to perform CAR design optimization, new CAR screening, and CAR safety investigation in a fast, efficient and safe manner, including in clinical trials. Nevertheless, the short-term expression of CAR molecule on the cell surface requires repeated injection of mRNA CAR-modified T cells. Therefore, an efficient immune cell expansion system is important to produce the large number of activated γδ T cells and CIK cells required for adoptive transfer of mRNA CAR T cells.

Based on IFNγ release and cytotoxicity assays, we observed a relatively lower cytotoxic immune responses induced by CIKZ cells modified with 3rd generation CARs as compared with those modified with 2nd generation CARs, possibly due to the reduced electroporation efficiency related to the increased mRNA size of the 3rd generation CARs. Although the difference between anti-CD19 CARs incorporating the CD27 and CD28 costimulatory domains is not so evident, we selected CD28 CARs for further investigations in the current study since CD28 has been commonly tested in clinical trials. Our findings indicated that the specificity and strength of cytotoxicity of CIKZ cells could be significantly enhanced through genetic modification with mRNA CARs specific for tumor antigens, as demonstrated with anti-CD19, anti-CEA, and anti-HER2 CARs in the present study. The possibility of using CIKZ cells for adoptive CAR cell therapy warrants further preclinical and clinical evaluation.

In conclusion, the K562 feeder cell-based CIKZ expansion method reported in the current study is efficient in expanding both CIK cells and γδ T cells. The final expansion products possess a highly functional and non-exhausted phenotype and show a low CD4/CD8 ratio. Most importantly, our CIKZ protocol is able to co-expand the required number of γδ T cells and CIK cells in a short time and it employs a simpler and less costly production method. The expanded CIKZ cells are inherently cytotoxic to tumor cells and they are easy to be genetically modified by CAR constructs to meet further requirements for adoptive cancer immunotherapy.

## Supporting Information

S1 Fig Gating strategy for αβT, γδT, NK, CD8 and CD4 populations. Day 21 CIKZ cells were used as an example. Multicolor flow cytometry was applied to analyze various cell populations simultaneously.
(PDF)

S2 Fig Gating strategy for CD8 effector memory cells, Treg cells, and PD1-positive cells. Day 21 CIKZ cells were used as an example. Multicolor flow cytometry was applied to analyze various cell populations simultaneously.
(PDF)
