# Supplementary material for: Co-Expansion of Cytokine-Induced Killer Cells and Vγ9Vδ2 T Cells for CAR T-Cell Therapy
Source: PLoS One. 2016 Sep 6;11(9):e0161820. doi: 10.1371/journal.pone.0161820 (PMC5012695; doi:10.1371/journal.pone.0161820)

S1 Figure. Gating strategy for αβT, γδT, NK, CD8 and CD4 populations. Day 21 CIKZ cells were used as an example. Multicolor flow cytometry was applied to analyse various cell populations simultaneously.

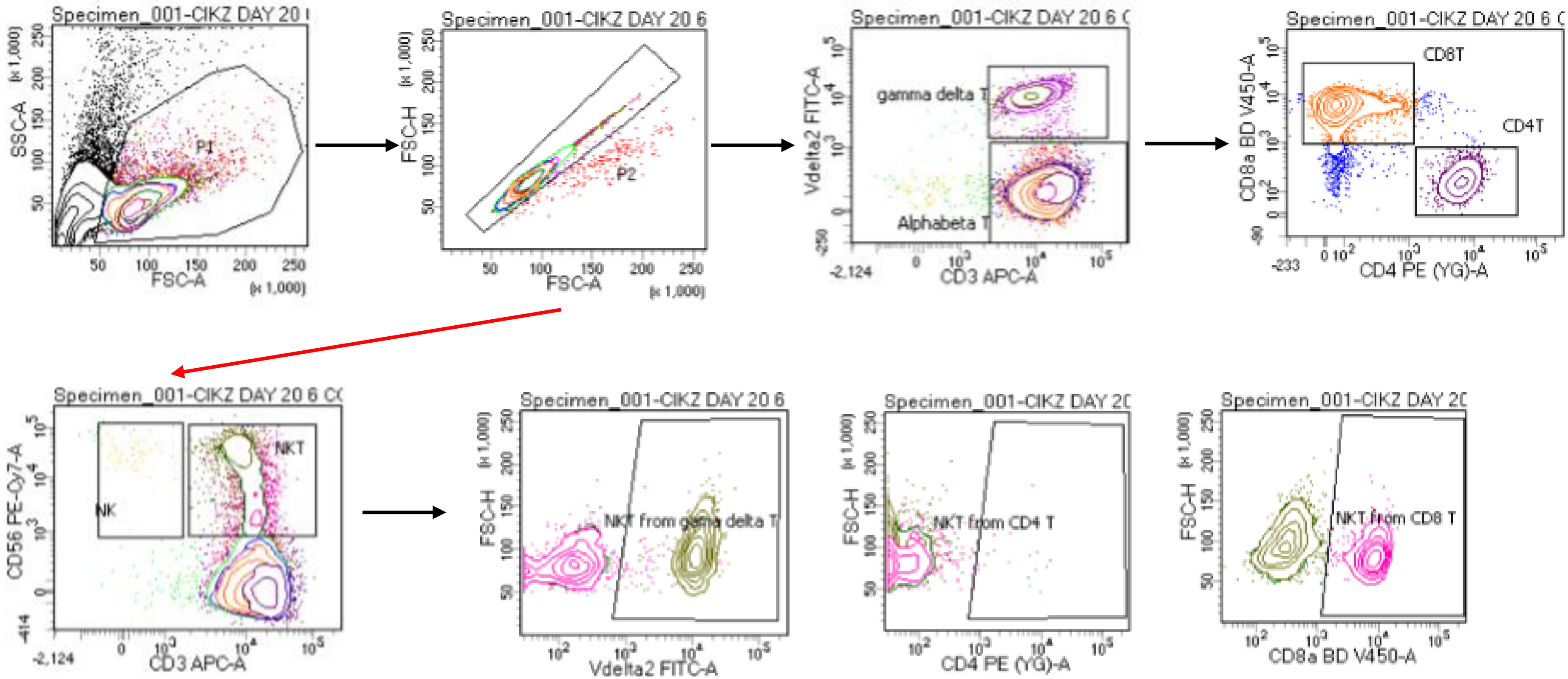

Supplement: S1 Fig — Day 21 CIKZ cells were used as an example. Multicolor flow cytometry was applied to analyze various cell populations simultaneously. (PDF) [file pone.0161820.s001.pdf]
