# Supplementary material for: Co-Expansion of Cytokine-Induced Killer Cells and Vγ9Vδ2 T Cells for CAR T-Cell Therapy
Source: PLoS One. 2016 Sep 6;11(9):e0161820. doi: 10.1371/journal.pone.0161820 (PMC5012695; doi:10.1371/journal.pone.0161820)

S2 Figure. Gating strategy for CD8 effector memory cells (A), Treg cells (B), and PD1-positive cells (C). Day 21 CIKZ were used as an example. Multicolor flow cytometry was applied to analyse various cell populations simultaneously.

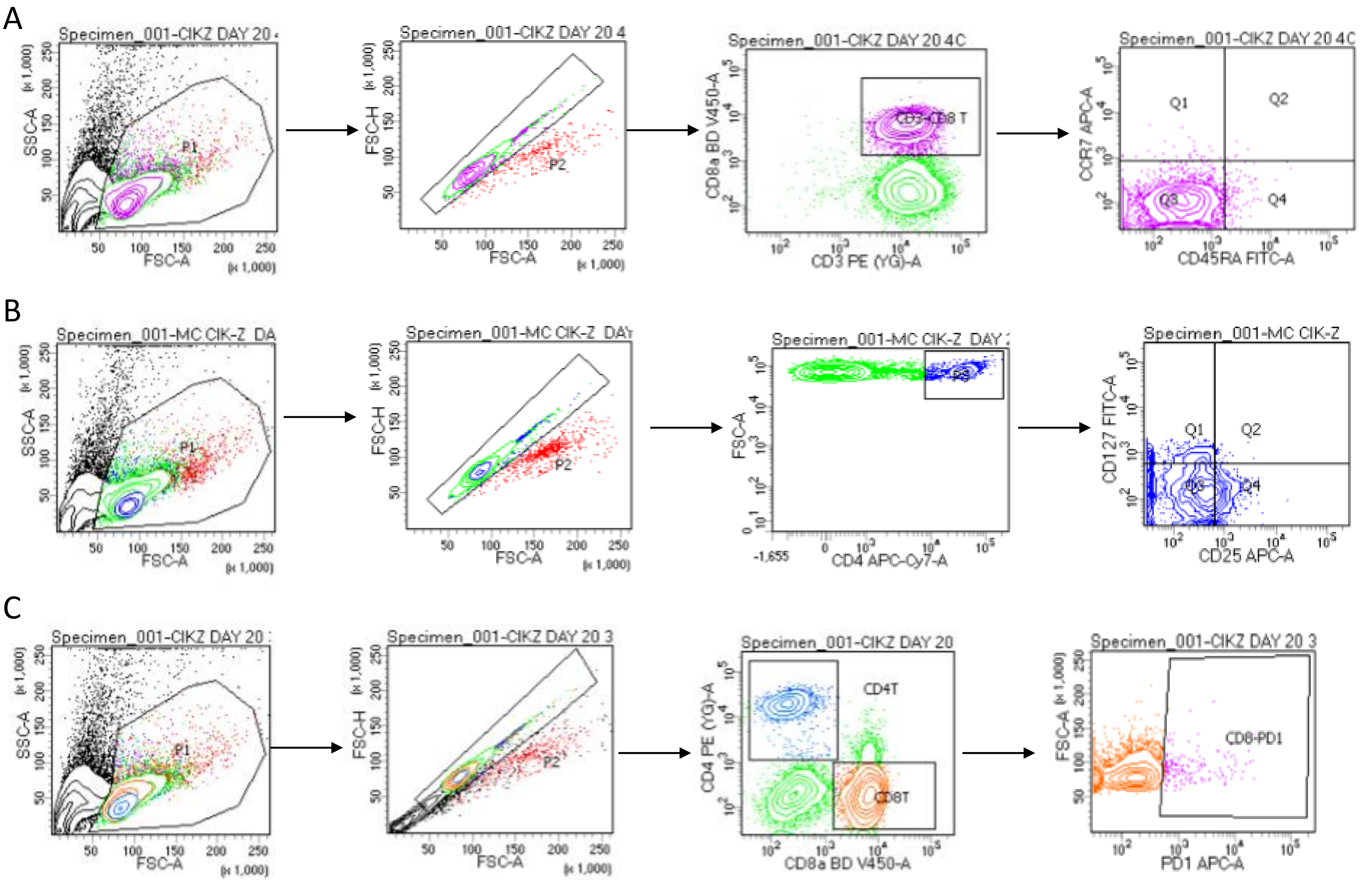

Supplement: S2 Fig — Day 21 CIKZ cells were used as an example. Multicolor flow cytometry was applied to analyze various cell populations simultaneously. (PDF) [file pone.0161820.s002.pdf]
